# Forest Owners' Response to Climate Change: University Education Trumps Value Profile

**DOI:** 10.1371/journal.pone.0155137

**Published:** 2016-05-25

**Authors:** Kristina Blennow, Johannes Persson, Erik Persson, Marc Hanewinkel

**Affiliations:** 1 Department of Landscape Architecture, Planning and Management, Swedish University of Agricultural Sciences, Alnarp, Sweden; 2 Department of Philosophy, Lund University, Lund, Sweden; 3 Chair of Forestry Economics and Forest Planning, University of Freiburg, Freiburg, Germany; Potsdam Institute for Climate Impact Research, GERMANY

## Abstract

Do forest owners’ levels of education or value profiles explain their responses to climate change? The cultural cognition thesis (CCT) has cast serious doubt on the familiar and often criticized "knowledge deficit" model, which says that laypeople are less concerned about climate change because they lack scientific knowledge. Advocates of CCT maintain that citizens with the highest degrees of scientific literacy and numeracy are not the most concerned about climate change. Rather, this is the group in which cultural polarization is greatest, and thus individuals with more limited scientific literacy and numeracy are more concerned about climate change under certain circumstances than those with higher scientific literacy and numeracy. The CCT predicts that cultural and other values will trump the positive effects of education on some forest owners' attitudes to climate change. Here, using survey data collected in 2010 from 766 private forest owners in Sweden and Germany, we provide the first evidence that perceptions of climate change risk are uncorrelated with, or sometimes positively correlated with, education level and can be explained without reference to cultural or other values. We conclude that the recent claim that advanced scientific literacy and numeracy polarizes perceptions of climate change risk is unsupported by the forest owner data. In neither of the two countries was university education found to reduce the perception of risk from climate change. Indeed in most cases university education increased the perception of risk. Even more importantly, the effect of university education was not dependent on the individuals' value profile.

## Introduction

Do forest owners' levels of education or value profiles explain their responses to climate change? The cultural theory of risk [1] and its recent offspring, the cultural cognition thesis (CCT) [2] have challenged the familiar and much discussed "knowledge deficit" model (e.g. [3,4]) in which scientific education plays an important role in fostering understanding in people who can learn about and adapt their decision-making to new information, including information about changing climate. Scientific literacy and numeracy are positively correlated with education level [5]; thus, it is plausible that the CCT might explain part of the dynamics of education level and value profile impact on forest owners' responses to climate change. According to CCT an individual's beliefs will converge with those of people with whom he or she shares common values. One implication of CCT is consistent with the claims of [6], which reports that people with a lower education level perceive higher risk than those with a higher education level. However, this depends on whether the situation is "pathological" [7]. The CCT claims that in "pathological" situations complex psychological mechanisms reflecting the segment of the public to which an individual belongs make him or her adopt interpretations of scientific evidence and perceptions of risk that fit the world view he or she has [2]. Science-literate individuals often become more culturally polarized because they have the specific capacity to search out and interpret evidence in patterns that sustain the convergence between their risk perceptions and their group identities [7]. In this paper we assess this application of CCT. In particular, we ask whether, under certain circumstances at least, climate change risk is indeed perceived to be higher by forest owners with lower levels of educational attainment (specifically, more limited scientific literacy and numeracy) than it is by forest owners with higher education levels. We expect to find that the influence of the cultural and other values of individuals on this relationship is statistically significant. If the psychological mechanisms identified by CCT operate among forest owners, we should observe polarizing differences in risk perception between groups of people with different value profiles. However, CCT claims that polarization occurs only in people with certain cultural values, and that the mechanisms are salient only in certain "pathological" [7] situations. Hence in what follows our findings relate to the applicability of CCT to forest owners specifically, not CCT as such. Notwithstanding that people also learn from sources in their environment, the knowledge deficit model suggests that education plays an important role in equipping people to address predicaments like climate change.

Forests are directly exposed to, and dependent on, the climate. Thus, forest owners who assign value to their forests and therefore have a stake in climate change are likely to be highly sensitive to the issue of climate change. Previous research has found that Swedish and German forest owners' perceptions of the risks posed by climate change differ widely, and that the variation can be explained almost completely by individual experiences of the effects of climate change and the strength of an individual's belief in local climate change effects [8,9]. Other studies support the significance of these two factors in perceptions of climate change risk [10] and have found that they are interdependent [11].

Thus, in order to study the dynamics of the impact of education level and value profile on forest owners' responses to climate change, and in an attempt to test the applicability of the CCT to these dynamics, we designed a questionnaire study to assess preferences for various services and benefits provided by the participant's forest, perceptions of climate change risk, and education level among private forest owners in Sweden and Germany. The countries were chosen so as to include forest owners operating in different economic, social, political and cultural structures in Europe. The reported strengths of belief in the local effects of climate change and personal experience of the effects of climate change were used as measures of perceived risk from climate change [8,9]; and reported levels of educational attainment were used to divide respondents into those who had, and those who had not, studied at university, thus mimicking high and low levels of scientific literacy and numeracy, respectively. Although the correlation between science literacy and numeracy and educational level is not perfect, this positive correlation is well established (e.g. [5]). The data obtained were used to test the hypothesis that under certain circumstances the forest owner's perception of risk from climate change correlates negatively with educational level and depends on valuations.

## Materials and Methods

We designed a questionnaire study to assess the preferences for services and benefits provided by the forest and perceptions in relation to climate change of 1,335 private forest owners in Sweden and Germany. The questionnaire data were sufficient for multivariate analysis [12]. The questions explored the forest owners' preferences for 95 services and benefits provided by their own forests, their personal beliefs in the local effects of climate change and whether they had experienced climate change and/or its consequences. Forest owners were also asked what their highest level of education was (Table 1 and S1 Table).

**Table 1:** Questions assessing respondents’ risk perceptions relating to climate change and education, and response options.

*Question*	*Abbreviation used in the text*	*Response options*
1. Do you believe that the climate is changing to such an extent that it will substantially affect your forest?	Do you believe in local effects of climate change?	Yes, definitely
		Yes, probably
		Do not know
		Probably not
		Definitely not
2. Have you experienced any extreme weather conditions or change in climate that you interpret as caused by long-term, global climate change?	Have you experienced effects of climate change?	Yes, definitely
		Yes, probably
		Do not know
		Probably not
		Definitely not
3. What education do you have?		Elementary school or equivalent
		High school or equivalent
		Professional education or equivalent
		University education or equivalent
		Professional education or equivalent and University education or equivalent

The questionnaire was formulated in English and translated into the native language of the respondents in each country. The Swedish forest owners were randomly sampled from contact people with forest holdings larger than 5 ha who were listed in the Swedish Real Property Register (Swedish Act 2000:224). Each recipient was assigned a code to enable targeted reminders to be sent to those who did not reply. To allow researchers to connect a particular answer to a particular respondent, the file containing responses needed to be cross-tabulated, which has not been done at any time. In Germany, the questionnaire was sent to all members of the forest owner organization Forstkammer Baden-Württemberg. The dispatch of these questionnaires was facilitated by this organization and the authors of this study had no access to identifying information for these individuals nor did they collect such information. The questionnaires were distributed by mail in spring 2010, accompanied by a covering letter explaining the objectives of the study and the purpose for which the data collected would be used. Respondents returned the questionnaires voluntarily as described in detail previously [8,13].

The research adhered to Swedish law on research involving human participants (Swedish Act 2003:460) and the handling of personal data (Swedish Act 1998:204). No further approval by the authors´ equivalent to the institutional review board (Etikprövningsnämnden) was necessary as described in detail previously [8]. This was also confirmed for this study by a representative of the Etikprövningsnämnden. The data are archived at the Swedish University of Agricultural Sciences, and access to them is regulated by Swedish secrecy legislation (The Personal Data Act, 1998:204, and the Public Access to Information and Secrecy Act 2009:400). The research material can be accessed by anyone with a legitimate interest in it. Requests should be addressed to the corresponding author.

A total of 786 forest owners returned the questionnaire (response rate 58.9%). Additional responses collected from Portuguese forest owners were excluded from this study owing to a lower response rate. Details of the data collection procedure and of quality control measures are described in [13]. The responses of the 766 forest owners who responded to questions about their preferences for forest services and benefits (S1 and S2 Tables) were used.

**Fig 1:**
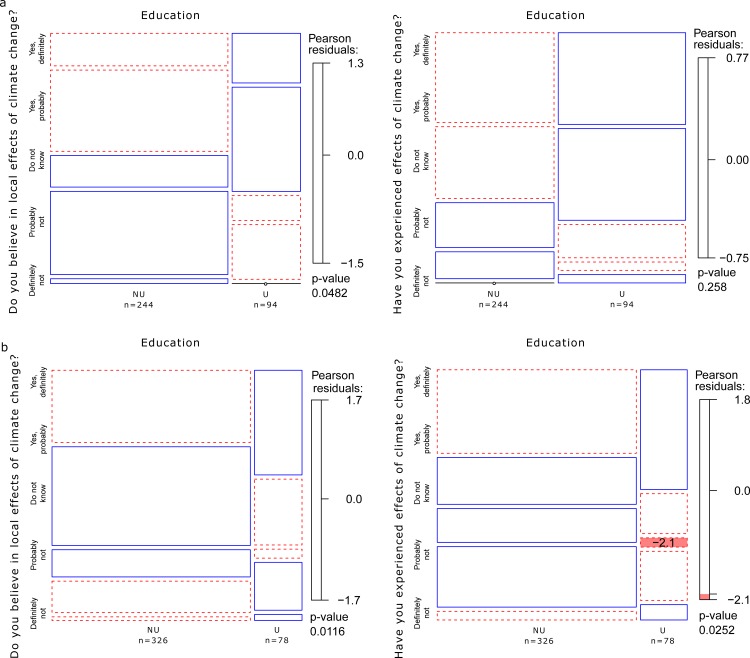
Relationship of climate change risk perception with university education. Relationships of risk perception in terms of the strength of belief in the local effects of climate change, the strength of belief in having experienced the effects of climate change and university education for Swedish (a) and German (b) respondents. The size of the respective compartment is proportional to the number of observations in the respective category. Pearson residuals outside of ±2 correspond to a significant difference for individual cells at approximately α = 0.05. Positive Pearson residuals are delineated in blue and negative residuals in red. The graphs are based on raw data before imputation. NU–No university education; U–University education.

**Fig 2:**
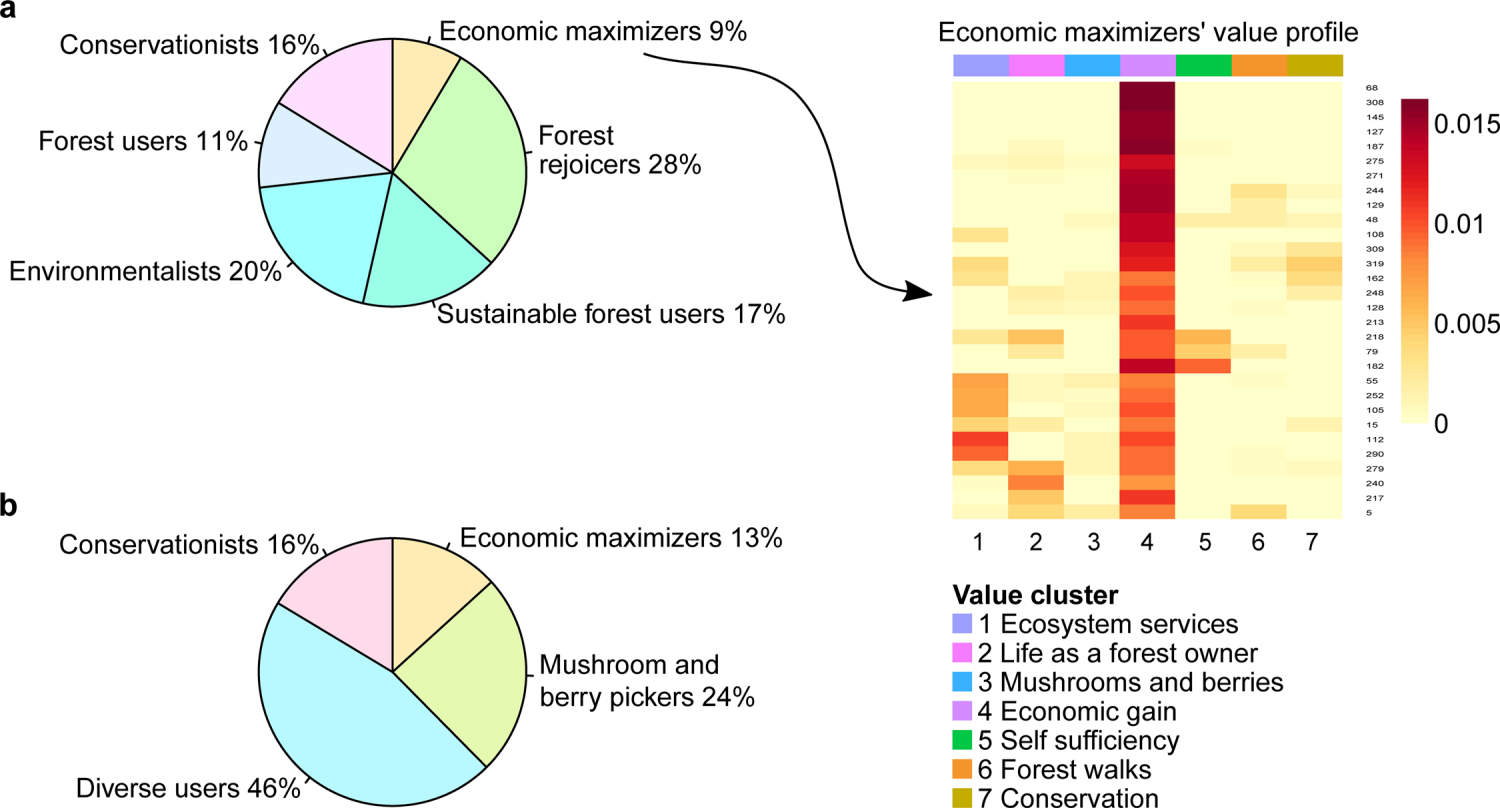
Value profiles and percentage of respondents by country. Value profiles for identified groups in Sweden (a) and Germany (b) based on individual respondents’ preference loadings (S2 Fig) on all value clusters identified in each country (S1 Fig). Inserted example shows loadings on value clusters for the 30 Swedish respondents with an "Economic maximizer" value profile.

The Pearson's χ^2^-test with simulated p value [14] was used to test for differences between groups of data (Fig 1). Preferences for services and benefits were reported on a scale from 0 to 10, with 0 denoting no value and 10 denoting the highest value (with missing data for individual questions interpreted as 0). In valuations of this kind, respondents are known to use scales of measurement that often are non-linear and that differ between individuals [15]. The individuals' valuations for each country were optimally scaled to maximize the sum of the largest eigenvalues [16] for each country (S3 Table), the number of which was determined using scree plots. The optimally scaled transformations (>0) were then used for each country to co-cluster the benefits, services and respondents using the machine-learning technique of non-negative matrix factorization (NMF) (Lee and Seung 1999), to identify clusters of value items and show how the forest owners' preferences were loaded on these (S2 and S3 Figs). Non-negative matrix factorization has previously been successfully used for feature recognition in diverse fields of study (e.g. [17–18]). To enable consideration of different value strengths, the individuals’ loadings on the identified clusters of values were used to cluster private forest owners for each country into groups representing different value profiles using the Affinity Propagation Clustering methodology [19] (Fig 2, S3 Fig and Table 2). The data for each country were analyzed separately so as not to make any assumptions about the cross-national validity of the value profiles identified [20].

**Table 2:** Value profile interpretations per country.

*Country*	*Value profile*	*Interpretation*
Sweden	**Forest rejoicers**	Assign value to life as a forest owner, mushroom and berry picking and forest walks. The value that is least interesting for this group is conservation.
	**Sustainable forest users**	Primarily interested in extracting resources from the forest for their own use. Secondarily they are interested in conservation and ecosystem services.
	**Economic maximizers**	Almost exclusively interested in economic gain. All other values score low for this group.
	**Environmentalists**	Have much in common with Sustainable forest users. The most salient difference is that the Environmentalists have a low interest in self-sufficiency while this is the primary driver for the Sustainable forest users. Environmentalists are primarily driven by interest in ecosystem services and conservation.
	**Forest users**	The opposite of the Environmentalists´ value profile. The primary driver for Forest users is self-sufficiency while ecosystem services have a low priority.
	**Conservationists**	Primarily weakly focused on conservation.
Germany	**Mushrooms and berry pickers**	Primarily interested in mushrooms and berries–both the mushrooms and berries themselves and the activity of picking them.
	**Conservationists**	Primarily interested in the plants and animals of the forest. Mostly the interest takes the form of conservation, but it is also to some extent an interest in hunting.
	**Economic maximizers**	Primarily interested in economic gain from the forest.
	**Diverse users**	Do not have one clear interest in the forest. The slightly dominating values have to do with production and ecosystem services. The value that is least in focus for this group is conservation.

To manage missing data (S4 Table), we used questions about strengths of belief in the local effects of climate change and having experienced effects of climate change (which were taken to represent the perception of risk from climate change [8,9]), preferences clustered into value profiles, and educational level as variables to infer five complete data sets (n = 766) using maximum likelihood methodology [21] (S4 Fig). After ensuring that the tentative variables passed a test for collinearity based on the variance inflation factor [22], we applied multinomial logistic regression to all five datasets in each country to test for differences between the groups that were differentiated by level of education and value profile with regard to their stated strength of belief in the local effects of climate change and having experienced the effects of climate change. The best and most parsimonious models were chosen by backward selection after adding all variables using Akaike's Information Criterion (AIC) as a performance indicator. The expected probabilities of the respondents' strengths of belief in the local effects of climate change and in having experienced the effects of climate change were estimated from 25,000 simulations drawn from the posterior distribution for each model. All analyses were conducted using the R Project for Statistical Computing packages v2.14.1 and v3.1.2 [23], and in particular by applying the libraries "vcd" for visualizing categorical data [24], "Aspect" for optimal scaling [25], "NMF" for nonnegative matrix factorization [26], "APCluster" for Affinity Propagation Clustering [27], "Amelia II" for multiple imputation [28], and "Zelig" for multinomial logistic regression modelling [29].

## Results

In neither of the two countries was university education found to reduce the perception of risk from climate change (S5–S8 Tables and Tables 3–7). Indeed in most cases university education increased the perception of risk. Even more importantly, the effect of university education was not dependent on the individuals' value profile. The perception of risk in terms of the strength of belief in the local effects of climate change was higher for Swedish and German respondents with university education than for those without (Tables 3 and 5 and S5 and S6 Tables). German respondents' value profiles did not correlate significantly with reported strengths of belief in the local effects of climate change (S6 Table). Strength of belief in the local effects of climate change was significantly stronger for Swedish respondents with an "Environmentalist" value profile than it was for those with other value profiles (Table 4), but there was no statistically significant interaction between the education and value profile variables. The "Environmentalist" value profile is driven by ecosystem services to a significant extent (Table 2, S3 Fig), which indicates high scientific literacy, a finding that provides a non-value-based explanation of the correlation.

**Table 3:** Predicted probabilities for strengths of belief in the local effects of climate change based on no university education and value profile, and relative risk ratios for strengths of belief in the local effects of climate change based on the education level (have/have not university education) and value profile using the model for Swedish respondents (S5 Table).

*Dependent variable*	*Response level*	*Mean probability (NU)*	*Mean probability SD*	*Mean risk ratio (U/NU)*	*Mean risk ratio SD*	*2*.*5%*	*97*.*5%*	*Statistically significant effect of U*
Do you believe that the climate is changing to such an extent that it will substantially affect your forest?	Value profile E: Yes, definitely	0.24	0.05	1.4	0.3	0.9	2.1	
	Value profile E: Yes, probably	0.43	0.06	1.1	0.2	0.8	1.5	
	Value profile E: Do not know	0.09	0.04	0.8	0.3	0.3	1.5	
	Value profile E: Probably not/Definitely not	0.24	0.06	0.6	0.1	0.3	0.9	*
Do you believe that the climate is changing to such an extent that it will substantially affect your forest?	Any value profile except E: Yes, definitely	0.18	0.06	2.5	1.0	1.1	5.1	*
	Any value profile except E: Yes, probably	0.44	0.07	1.0	0.3	0.5	1.6	
	Any value profile except E: Do not know	0.10	0.05	0.7	1.0	0.1	3.4	
	Any value profile except E: Probably not/Definitely not	0.27	0.07	0.4	0.3	0.1	1.1	

NU–No university education; U–University education; E–Environmentalists. The tests were based on 25,000 simulations drawn from the posterior distribution while keeping the education level constant, at university education and no university education, respectively, and made at α = 0.05.

* denotes statistically significant.

**Table 4:** Predicted probabilities for strengths of belief in the local effects of climate change based on value profile, and relative risk ratios for strengths of belief in the local effects of climate change based on value profile (with the education level represented by its proportion of those having studied at university) using the model for Swedish respondents (S5 Table).

*Dependent variable*	*Response level*	*Mean probability (any value profile except E)*	*Mean probability SD*	*Mean risk ratio (E/ other value profile)*	*Mean risk ratio SD*	*2*.*5%*	*97*.*5%*	*Statistically significant effect of value profile E*
Do you believe that the climate is changing to such an extent that it will substantially affect your forest?	Yes, definitely	0.14	0.02	1.9	0.5	1.1	3.0	*
	Yes, probably	0.36	0.03	1.2	0.2	0.9	1.6	
	Do not know	0.14	0.02	0.6	0.3	0.2	1.2	
	Probably not/Definitely not	0.36	0.03	0.6	0.1	0.3	0.9	*

E–Environmentalists. The tests were based on 25,000 simulations drawn from the posterior distribution while keeping the education level constant, at university education and no university education, respectively, and made at α = 0.05.

* denotes statistically significant.

**Table 5:** Predicted probabilities for strengths of belief in the local effects of climate change based on no university education and relative risk ratios for strengths of belief in the local effects of climate change based on education level (have/have not university education) among German respondents (S6 Table).

*Dependent variable*	*Response level*	*Mean probability (NU)*	*Mean probability SD*	*Mean risk ratio (U/NU)*	*Mean risk ratio SD*	*2*.*5%*	*97*.*5%*	*Statistically significant effect of U*
Do you believe that the climate is changing to such an extent that it will substantially affect your forest?	Yes, definitely	0.30	0.03	1.4	0.2	1.0	1.9	*
	Yes, probably	0.42	0.03	0.7	0.1	0.5	1.0	*
	Do not know	0.12	0.02	0.4	0.3	0.1	1.1	
	Probably not	0.14	0.02	1.5	0.4	0.9	2.4	
	Definitely not	0.02	0.08	2	2	0	8	

NU–No university education; U–University education. The tests were based on 25,000 simulations drawn from the posterior distribution while keeping the education level constant, at university education and no university education, respectively, and made at α = 0.05.

* denotes statistically significant.

**Table 6:** Predicted probabilities for strengths of belief in having experienced the effects of climate change based on value profile and relative risk ratios for strengths of belief in having experienced the effects of climate change based on value profile (Forest users/other) among Swedish respondents (S7 Table).

*Dependent variable*	*Response level*	*Mean probability (FR*, *SFU*, *EM*, *E*, *C)*	*Mean probability SD*	*Mean risk ratio (FU/other)*	*Mean risk ratio SD*	*2*.*5%*	*97*.*5%*	*Statistically significant effect of value profile FU*
Have you experienced any extreme weather conditions or change in climate that you interpret as caused by long-term, global climate change?	Yes, definitely	0.10	0.02	0.9	0.5	0.2	2.2	
	Yes, probably	0.18	0.02	1.5	0.4	0.8	2.5	
	Do not know	0.20	0.02	1.7	0.4	1.0	2.7	
	Probably not	0.47	0.03	0.5	0.1	0.2	0.8	*
	Definitely not	0.05	0.01	2	1	1	6	

FR–Forest Rejoicers; SFU–Sustainable Forest Users; EM–Economic Maximizers, E–Environmentalists; FU–Forest Users; C–Conservationists. The tests were based on 25,000 simulations drawn from the posterior distribution while keeping the value profile constant, at Forest users’ value profile and otherwise, respectively, and made at α = 0.05.

* denotes statistically significant.

**Table 7:** Predicted probabilities for strengths of belief in the local effects of climate change based on no university education and relative risk ratios for strengths of belief in having experienced the effects of climate change based on education level (have/have not university education) among German respondents (S8 Table).

*Dependent variable*	*Response level*	*Mean probability (NU)*	*Mean probability SD*	*Mean risk ratio (U/NU)*	*Mean risk ratio SD*	*2*.*5%*	*97*.*5%*	*Statistically significant effect of U*
Have you experienced any extreme weather conditions or change in climate that you interpret as caused by long-term, global climate change?	Yes, definitely	0.35	0.03	1.4	0.2	1.1	1.8	*
	Yes, probably	0.20	0.02	0.9	0.2	0.5	1.4	
	Do not know	0.15	0.02	0.3	0.2	0.1	0.8	*
	Probably not	0.26	0.02	0.8	0.2	0.5	1.2	
	Definitely not	0.04	0.01	1.8	1.0	0.6	4.2	

NU–No university education; U–University education. The tests were based on 25,000 simulations drawn from the posterior distribution while keeping the education level constant, at university education and no university education, respectively, and made at α = 0.05.

* denotes statistically significant.

Strength of belief in having experienced the effects of climate change was correlated with university education for German but not Swedish respondents (S7 and S8 Tables and Tables 6 and 7). This component of the perception of climate-change risk was correlated with the value profiles for respondents in Sweden (Table 6). Swedish respondents with any value profile except "Forest user" reported that they had not experienced the effects of climate change (i.e. a response of "Probably not") significantly more often than those with the "Forest user" value profile (Table 6). The "Forest user" value profile is driven primarily by self-sufficiency (Table 2), which indicates that those in this group spend considerable amounts of time in the forest, a finding that provides a non-value-based explanation of the observed difference.

## Discussion and Conclusions

The results show that the dynamics between valuations, educational level, and risk perception predicted by CCT are not at work in the domain investigated in this study. While this result is far from a refutation of the CCT as such, it does show that the CCT has no explanatory power in connection with the climate change responses among forest owners in Sweden and Germany. Furthermore, it provides information that is valuable in its own right in that it helps for understanding forest owner climate change response.

Other factors may contribute to the explanation of the differences between the results in [2] and the results presented in this study. It is possible that the combined measure of scientific literacy and numeracy that Kahan et al. [2] construct is not useful for predicting differences between people with different educational levels. Additionally, the methods used in our study account for particularities associated with the analysis of data on individual scales of measurement; Kahan et al. [2] assumed that the data fell on an interval scale to justify the use of linear regression methodology. Thus, in [2] the use of linear regression methodology on rating scale and count data might have significantly affected the results.

We conclude that the results do not converge with those that would be expected if the mechanisms identified by the CCT were in play, and hence we find no evidence that forest owner value profiles exert a stronger influence on risk perception than university education does. This result is important for the design of effective strategies to engage forest owners to respond to climate change. While [2] suggests that climate change information should be adapted to the audience's valuations to be effective, the results in the present study imply that in most cases there is no reason to rule out education as a means of fostering understanding in forest owners who can learn about and adapt their decision-making to a changing world.

## Supporting Information

S1 Fig Value clusters by country. Clusters among 95 value items (numbers below each column correspond to questions in S1 Table) identified for optimally scaled valuations made by respondents in Sweden (a) and Germany (b) across 500 runs for each country, respectively.
(TIF)

S2 Fig Loadings on value items by country. Loadings on value items estimated for valuations made by respondents (rows) in Sweden (a) and Germany (b) across 500 runs for each country, respectively.
(TIF)

S3 Fig Value profiles by country. Value profiles for identified groups in Sweden (a) and Germany (b) based on individual respondents' preference loadings (S2 Fig) on all value clusters identified in each country (S1 Fig), respectively. Boxes denote the interquartile range, and whiskers extend to the minimum and maximum data points while the bold horizontal line indicates the median. Elaborate interpretations of the value profiles are provided in Table 2.
(TIF)

S4 Fig Relationship of climate change risk perception with tentative explanatory variables. Relationships of the belief in the local effects of climate change and having experienced the effects of climate change, taken as representing components of the perception of climate change risk, highest education level and value profile for Swedish (a) and German (b) respondents. The size of the respective compartment is proportional to the number of observations in the respective category. The graphs are based on data after imputation.; FR–Forest Rejoicers; SFU–Sustainable Forest Users; EM–Economic Maximizers; E–Environmentalists; FU–Forest Users; C–Conservationists; MBP–Mushroom and Berry Pickers; DU–Diverse Users.
(TIF)

S1 File Questionnaire. (PDF)

S1 Table Questions assessing respondents' preferences for 95 services and benefits from the forest and the range and median score (0–10) assigned by respondents who reported having not studied or studied at university, based on a question reporting respondents' highest level of education per country (see Table 1). (n = 766). (DOCX)

S2 Table Number of questionnaires distributed and returned with responses to the questions on preferences (S1 Table) per country. (DOCX)

S3 Table Number of clusters among respondents in scaling, and variance accounted for by these, by country. (DOCX)

S4 Table Missingness before imputation by question and country. (DOCX)

S5 Table Diagnostic statistics of model for predicting climate change risk perception in terms of strength of belief in the local effects of climate change by forest owners in Sweden based on education level and value profile. S.b. climate change—Strength of belief in the local effects of climate change, NU–No University education; U–University education; E–Environmentalists. The value profile Forest rejoicers was combined with Forest users and Sustainable forest users with Economic maximizers during model fitting because of quasi-complete separation (S4 Fig). The model was fitted to five imputed datasets using multinomial logistic regression. The mean null deviance = 910.6, the degrees of freedom for the null model = 1050, residual deviance = 888.5, and the residual degrees of freedom = 1044. The model fits the data significantly better than the null model (p = 0.0012).
(DOCX)

S6 Table Diagnostic statistics of model for predicting climate change risk perception in terms of strength of belief in the local effects of climate change by forest owners in Germany based on education level. S.b. climate change—Strength of belief in the local effects of climate change; NU–No University education; U–University education. The value profile Mushroom and berry pickers was combined with Diverse users and Conservationists with Economic maximizers during model fitting because of quasi-complete separation (S4 Fig). The model was fitted to five imputed datasets using multinomial logistic regression. The mean null deviance = 1093.8, the degrees of freedom for the null model = 1656, mean residual deviance = 1081.3, and the residual degrees of freedom = 1652. The model fits the data significantly better than the null model (p = 0.014).
(DOCX)

S7 Table Diagnostic statistics of model for predicting climate change risk perception in terms of strength of belief in having experienced the effects of climate change by forest owners in Sweden based on value profile. S.b. exp. climate change—Strength of belief in having experienced climate change; FU–Forest user value profile. The value profile Forest rejoicers was combined with Sustainable forest users and Economic maximizers with Conservationists during model fitting because of quasi-complete separation (S4 Fig). The model was fitted to five imputed datasets using multinomial logistic regression. The mean null deviance = 965.6, the degrees of freedom for the null model = 1400, the mean residual deviance = 954.3, and the residual degrees of freedom = 1396. The model fits the data significantly better than the null model (p = 0.024).
(DOCX)

S8 Table Diagnostic statistics of model for predicting climate change risk perception in terms of strength of belief in having experienced the effects of climate change by forest owners in Germany based on education level. S.b. exp. climate change—Strength of belief in having experienced climate change; NU–No University education; U–University education. The value profile Mushroom and berry pickers was combined with Conservationists and Economic maximizers with Diverse users during model fitting because of quasi-complete separation (S4 Fig). The model was fitted to five imputed datasets using multinomial logistic regression. The mean null deviance = 1189.0, the degrees of freedom for the null model = 1656, mean residual deviance = 1174.2, and the residual degrees of freedom = 1652. The model fits the data significantly better than the null model (p = 0.0051).
(DOCX)
