# Supplementary figures and images for: Forest Owners' Response to Climate Change: University Education Trumps Value Profile
Source: PLoS One. 2016 May 25;11(5):e0155137. doi: 10.1371/journal.pone.0155137 (PMC4880312; doi:10.1371/journal.pone.0155137)

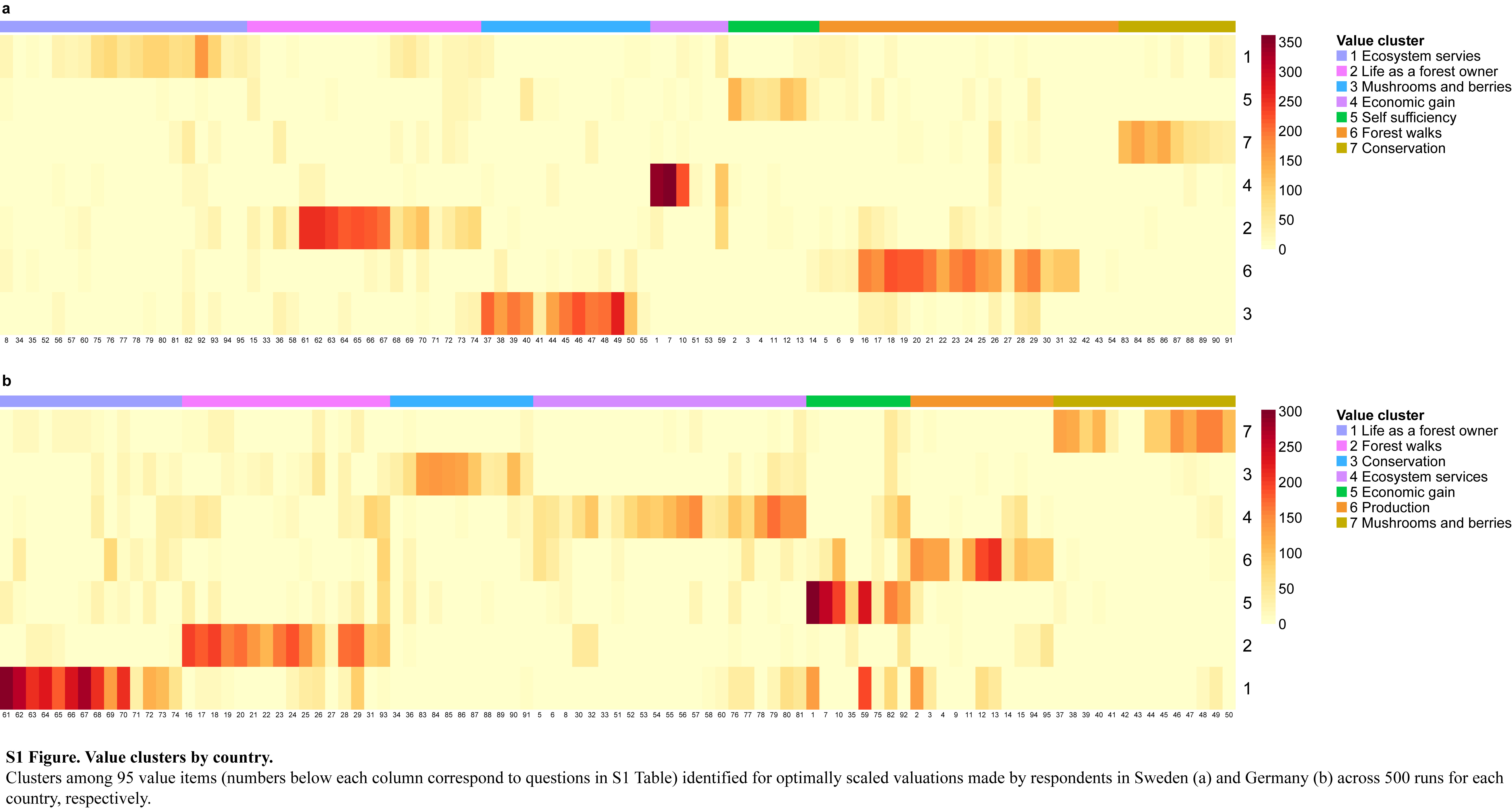

Supplement: S1 Fig — Clusters among 95 value items (numbers below each column correspond to questions in S1 Table) identified for optimally scaled valuations made by respondents in Sweden (a) and Germany (b) across 500 runs for each country, respectively. (TIF) [file pone.0155137.s001.tif]

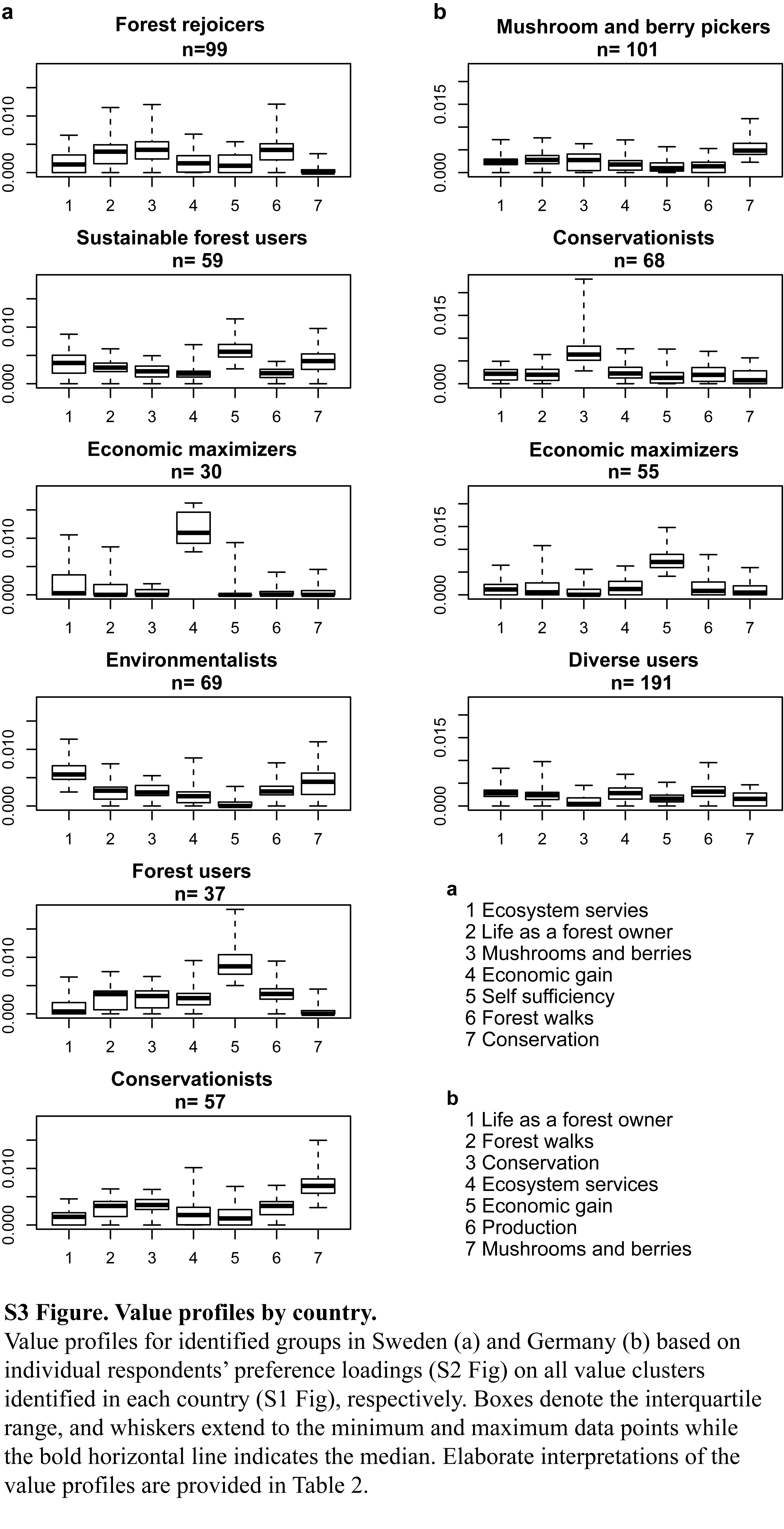

Supplement: S3 Fig — Value profiles for identified groups in Sweden (a) and Germany (b) based on individual respondents' preference loadings (S2 Fig) on all value clusters identified in each country (S1 Fig), respectively. Boxes denote the interquartile range, and whiskers extend to the minimum and maximum data points while the bold horizontal line indicates the median. Elaborate interpretations of the value profiles are provided in Table 2. (TIF) [file pone.0155137.s003.tif]

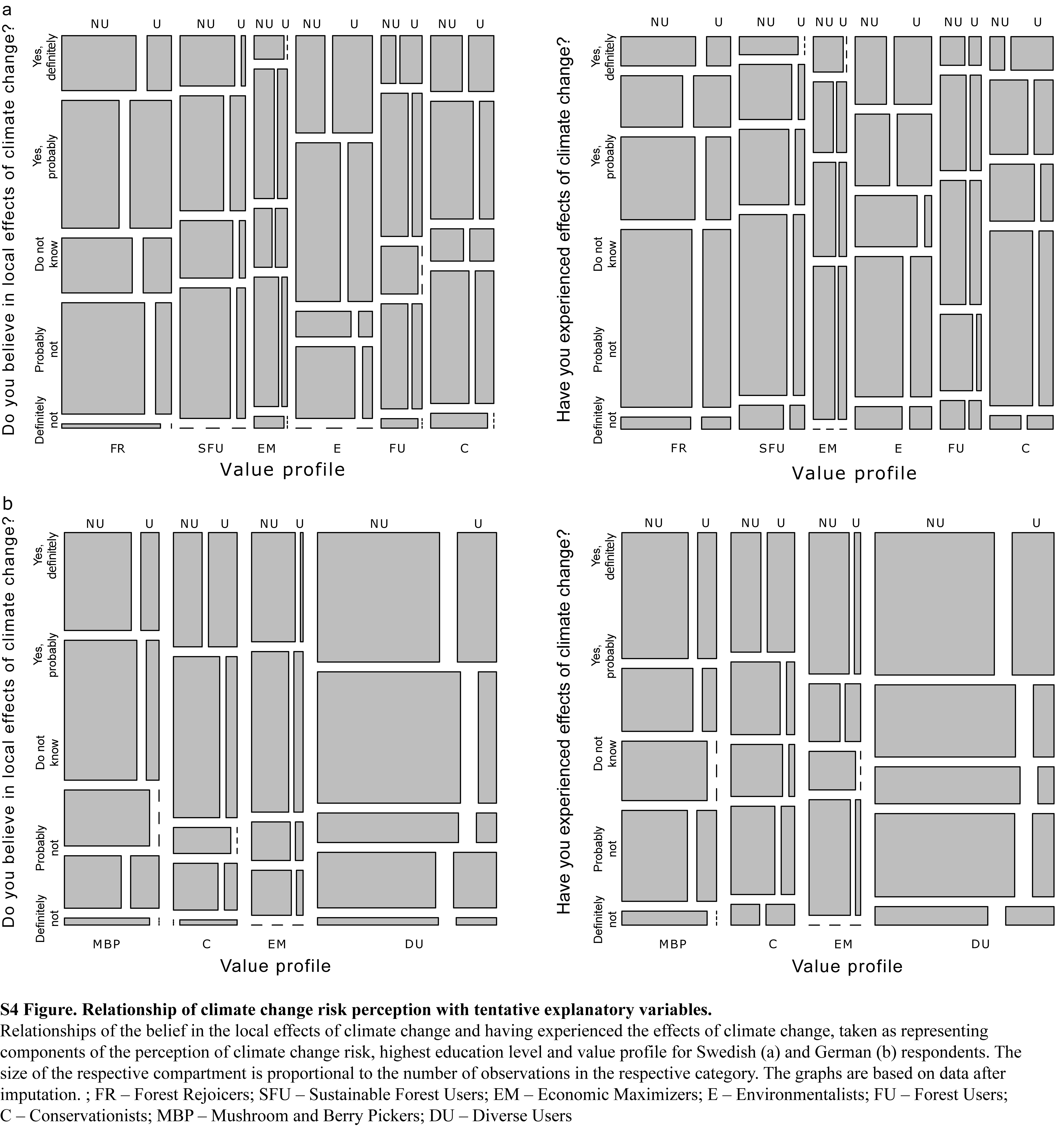

Supplement: S4 Fig — Relationships of the belief in the local effects of climate change and having experienced the effects of climate change, taken as representing components of the perception of climate change risk, highest education level and value profile for Swedish (a) and German (b) respondents. The size of the respective compartment is proportional to the number of observations in the respective category. The graphs are based on data after imputation.; FR–Forest Rejoicers; SFU–Sustainable Forest Users; EM–Economic Maximizers; E–Environmentalists; FU–Forest Users; C–Conservationists; MBP–Mushroom and Berry Pickers; DU–Diverse Users. (TIF) [file pone.0155137.s004.tif]
