# Supplementary material for: Forest Owners' Response to Climate Change: University Education Trumps Value Profile
Source: PLoS One. 2016 May 25;11(5):e0155137. doi: 10.1371/journal.pone.0155137 (PMC4880312; doi:10.1371/journal.pone.0155137)
Supplement: S1 File — (PDF) [file pone.0155137.s005.pdf]

First a few questions about you and your property

1. **My answers concerns the management unit* in the municipality of** ____________________

   * Enter the definition of a management unit for Germany/Portugal here!

2. **What is the acreage of your management unit?**

   Approximately ___________ hectares

3. **What is the acreage of land for different land uses on your management unit?**

   ❑ Forest land ⇨ Approximately ___________ hectares

   ❑ Grazing land ⇨ Approximately ___________ hectares

   ❑ Crop land ⇨ Approximately ___________ hectares

   ❑ Other land use, what? __________________ approximately _________ hectares

4. **Do you farm all of the grazing land and farm land yourself?**

   ❑ Yes
   ❑ No ⇨ Approximately ___________ hectares of grazing land is leased out

   ⇨ Approximately ___________ hectares of crop land is leased out

   ⇨ Approximately ___________ hectares are not managed actively

5. **Are you the sole owner of the management unit or do you own it together with other persons?**

   ❑ I am the sole owner of the management
   ❑ I own the management unit together with other persons

   ⇨ We are _______ owners (including myself)

6. **What year did you become the owner/joint owner of the management unit?**

   Year ___________

7. **How did you become the owner of the management unit?**

   ❑ Heritage or gift
   ❑ Bought it from a family member or relative
   ❑ Bought it from another person/organization/company

8. Do you live on the management unit?

(mark with one cross)

- ❑ Yes
- ❑ Yes, but I am planning to move from the management unit
- ❑ No
- ❑ No, but I am planning to move to the management unit

If you answered no, how far is it between the management unit and your home?

Approximately ______ kilometers

9. What kind of relationship did you have to forest and forestry when you grew up?

- ❑ I grew up on the management unit
- ❑ I spent a lot of time on the management unit during my childhood
- ❑ I worked on the management unit
- ❑ I worked on another management unit
- ❑ I lived on another management unit
- ❑ I lived on the countryside or in a small town that had connections to forestry
- ❑ I lived on the countryside or in a small town that had no connections to forestry
- ❑ I lived in a city or bigger town that had connections to forestry
- ❑ I lived in a city or bigger town without connections to forestry

10. How often do you visit the management unit on average?

(mark with one cross)

- ❑ More than 3 times per week
- ❑ 1-3 times per week
- ❑ 1-2 times per month
- ❑ 2-4 times per year
- ❑ 1-2 times per year
- ❑ Less than one time per year

11. How is the responsibility mainly distributed when it comes to decisions regarding the forestry on the management unit?

(mark with one cross)

- ❑ I am making the decisions alone
- ❑ My husband/wife is making the decisions alone
- ❑ My husband/wife and I are making the decisions together
- ❑ A person (outside the household) is making the decisions alone
- ❑ A person (outside the household) and I are making the decisions together
- ❑ Husband/wife and another person are making the decisions together

12. Who do you think will take over the management unit in the future?

(mark with one cross)

☐ My own children
☐ Another close relative
☐ Another person than a relative
☐ No opinion/it is not relevant right now

13. Do you carry out any forestry operations (pre-commercial thinning, thinning, harvesting etc.) by yourself on your management unit?

☐ Yes ⇨ Approximately ____________ workdays per year

☐ No, but a family member/relative does ⇨ Approximately ____________ workdays per year

☐ No, I hire a forest company/contractor for all forestry operations

14. How large share of the forest acreage of your management unit is conifers and broadleaves, respectively?

100 % conifers<br>0 % broadleaves	50 % conifers<br>50 % broadleaves	0 % conifers<br>100 % broadleaves

15. How do you wish that the forest on your management unit will develop in the future?

☐ More spruce forest
☐ More mixed forest
☐ More exotic tree species (examples…)
☐ Smaller departments (forest stands)
☐ More pine forest
☐ More broadleaved forest
☐ Larger departments (forest stands)
☐ No change

☐ Other, how? ______________________________

16. If you wish to change the tree species composition on your management unit, what is the main reason?

(mark with one cross)

☐ Yes, because of expected climate change effects
☐ Yes, because of new goals with my/our forestry
☐ Yes, because I think that the wood market will change
☐ Yes, because I think a different composition will seize the growing opportunities better
☐ Yes, because I think that my private financial situation will change
☐ No, because I am satisfied with the tree species composition on my management unit
☐ No, because it is not possible to change the current tree species composition (by practical reasons)
☐ No, because I do not have enough knowledge about what tree species I can change to

☐ Other reason: ______________________________________

A few questions about risks and climate change

17. What is the risk of financial consequences for you and your household because of the following events?

(mark with one cross for each event)

**Events**	No risk	Low risk	High risk	Very high risk
Root rot damages	❑	❑	❑	❑
Bark beetle damages	❑	❑	❑	❑
Pine weevil damages	❑	❑	❑	❑
Browsing damages	❑	❑	❑	❑
Storm damages	❑	❑	❑	❑
Frost damages	❑	❑	❑	❑
Increased logging costs because of absence of ground frost	❑	❑	❑	❑
Snow damages	❑	❑	❑	❑
Drought damages	❑	❑	❑	❑
Flooding damages	❑	❑	❑	❑
Forest fire damages	❑	❑	❑	❑
Increased competition from ground vegetation	❑	❑	❑	❑
Reduced revenues from forestry	❑	❑	❑	❑
Increasing interest rates	❑	❑	❑	❑
Increasing property taxes	❑	❑	❑	❑

18. How certain were you when you assessed the risks in question 17?

(mark with one cross for each event)

**Events**	Certain	Fairly certain	Fairly uncertain	Uncertain
Root rot damages	❑	❑	❑	❑
Bark beetle damages	❑	❑	❑	❑
Pine weevil damages	❑	❑	❑	❑
Browsing damages	❑	❑	❑	❑
Storm damages	❑	❑	❑	❑
Frost damages	❑	❑	❑	❑
Increased logging costs because of absence of ground frost	❑	❑	❑	❑
Snow damages	❑	❑	❑	❑
Drought damages	❑	❑	❑	❑
Flooding damages	❑	❑	❑	❑
Forest fire damages	❑	❑	❑	❑
Increased competition from ground vegetation	❑	❑	❑	❑
Reduced revenues from forestry	❑	❑	❑	❑
Increasing interest rates	❑	❑	❑	❑
Increasing property taxes	❑	❑	❑	❑

19. Rank only five (5) of the following risks from 1 to 5, where 1 is the risk that you are willing to pay the highest amount to reduce (by for example modified management or insurance)

Root rot damages	_____	Drought damages	_____
Bark beetle damages	_____	Flooding damages	_____
Pine weevil damages	_____	Forest fire damages	_____
Browsing damages	_____	Increased competition from ground vegetation	_____
Storm damages	_____	Reduced revenues from forestry	_____
Frost damages	_____	Increasing interest rates	_____
Increased logging costs because of absence of ground frost	_____	Increasing property prices	_____
Snow damages	_____		

20. Do you take any actions <u>today</u> specifically in order to reduce the risks below?

(mark with <u>one</u> cross for each event)

**Events**	No	Do not know	Yes	If yes, how?
Root rot damages	❑	❑	❑	______________
Bark beetle damages	❑	❑	❑	______________
Pine weevil damages	❑	❑	❑	______________
Browsing damages	❑	❑	❑	______________
Storm damages	❑	❑	❑	______________
Frost damages	❑	❑	❑	______________
Increased logging costs because of absence of ground frost	❑	❑	❑	______________
Snow damages	❑	❑	❑	______________
Drought damages	❑	❑	❑	______________
Flooding damages	❑	❑	❑	______________
Forest fire damages	❑	❑	❑	______________
Increased competition from ground vegetation	❑	❑	❑	______________
Reduced revenues from forestry	❑	❑	❑	______________
Increasing interest rates	❑	❑	❑	______________
Increasing property taxes	❑	❑	❑	______________

Now, we would like to ask you some questions relating to the storm/fire of year XXXX

21. How large was the standing volume on the management unit <u>before</u> the storm/fire (date)?

(State the number of cubicmetres)

__________ m^3sk __________ m^3fub

22. How many cubicmetres of wood was damaged on the management unit in the storm/fire (date)?

(State the number of cubicmetres)

__________ m^3sk __________ m^3fub

23. How has the storms/fires (date) during recent years affected your relationship towards owning forest?

(mark with <u>one</u> cross)

❑ I will continue to own forest in the future
❑ I will sell my management unit within 10 years because of other reasons than the storms/fires.
❑ Because of the storms/fires, I will sell the management unit within 1 year.
❑ Because of the storms/fires, I will sell the management unit within 10 years.
❑ I do not know/I have not decided

24. Was your forest insured against storm/fire damage at the time of the storm/fire (date)?

❑ Yes ❑ No

25. Is your forest insured against storm/fire damage today?

❑ Yes ❑ No⇨ Why not ____________________________

26. If your forest was insured, what are your experiences from the insurance <u>after</u> the storm/fire (date)?

(mark with <u>one</u> cross)

❑ I was not affected by the storm/fire
❑ I was affected, but I have not tried to get compensation from the insurance
❑ I was affected and I have positive experiences from the insurance
❑ I was affected and have neither positive, nor negative experiences from the insurance
❑ I was affected and have negative experiences from the insurance

27. How aware were you of the risk for damages by storm/fire before the storm/fire (date) and how aware are you today?

	Not aware at all				Very much aware
Before the storm/fire	❑	❑	❑	❑	❑
Today	❑	❑	❑	❑	❑

28. Were any specific forestry operations carried out before the storm/fire to reduce the risk of damages from storm/fire? Do you carry out any specific operations now, after the storm/fire?

(mark with one cross per row)

	Yes	No
Before the storm/fire	❑	❑
Today	❑	❑

29. Would you like to change your forest management due to recent storm/fire damages in "the case study area" (example, date)?

❑ No change
❑ I would like to convert grazing land/crop land to forest land
❑ I would like to convert forest land to grazing land/crop land
❑ I would like to focus more on game management rather than timber production
❑ I would like to focus more on nature conservation rather than timber production
❑ I would like to focus more on timber production rather than game management
❑ I would like to focus more on timber production rather than nature conservation

❑ Other, what? ______________________________

30. Do you think that the climate is changing to such an extent that it substantially will affect your forest?

(mark with one cross)

❑ Yes, certainly ❑ No, scarcely
❑ Yes, perhaps ❑ No, definitely not ❑ I do not know

31. Has the climate change debate affected your forest management?

❑ Yes ❑ No

32. If you answered yes to question 31, in what ways have you adapted your forest management?

- ☐ I have increased the share of broadleaves on my management unit
- ☐ I have increased the share of conifers on my management unit
- ☐ I have increased the share of mixed forest on my management unit
- ☐ I make sure to get the timber out early from the forest while the ground is still frozen
- ☐ I manage for more variation in stand structure, stand age, and silvicultural treatments
- ☐ I have increased/introduced new (exotic) tree species

- ☐ Other: ____

33. If you answered no to question 31, what is the main reason?

(mark with one cross)

- ☐ I have not thought about climate change and my own forest management
- ☐ I do not believe that the climate is changing
- ☐ I do not know how to modify my forest management
- ☐ I do not know how the climate is changing
- ☐ There is too much uncertainty as to whether the climate is changing
- ☐ There is too much uncertainty about how the climate is changing
- ☐ Too much uncertainty about what management measures reduce negative consequences of climate change
- ☐ Too much uncertainty about what management measures increase positive effects of climate change

34. What is your main source of information about climate change?

- ☐ My forestry advisor (example of forestry advisors)
- ☐ Relatives, neighbors or friends
- ☐ The former owner of my management unit
- ☐ The owner of a neighboring management unit
- ☐ The owner of a management unit that has many similarities with my management unit
- ☐ Books, journals, TV, radio, internet etc.

35. Do you think the climate changes will affect the financial situation in your forestry?

Very negatively	Somewhat negatively	Not at all	Somewhat positively	Very positively

36. How certain were you when you answered question 35?

Uncertain — Certain

37. Have you experienced any extreme weather conditions that you interpret as caused by a long-term and global climate change?

(mark with one cross)

- ❑ Yes, certainly ➪ How? _______________________________
- ❑ Yes, perhaps ➪ How? _______________________________
- ❑ No, scarcely
- ❑ No, definitely not
- ❑ Do not know

38. Do you and your forestry advisor discuss management options that could:

	Yes	No
Reduce the negative effects of climate change	❑	❑
Increase the benefits from the positive effects of climate change	❑	❑

39. How do you believe climate change affects the risk of financial consequences for you and your household because of the events listed below?

(mark with one cross for each event)

**Events**	Much lower risk than today	Somewhat lower risk than today	The same risk as today	Somewhat higher risk than today	Much higher risk than today
Root rot damages	❑	❑	❑	❑	❑
Bark beetle damages	❑	❑	❑	❑	❑
Pine weevil damages	❑	❑	❑	❑	❑
Browsing damages	❑	❑	❑	❑	❑
Storm damages	❑	❑	❑	❑	❑
Frost damages	❑	❑	❑	❑	❑
Increased logging costs because of absence of ground frost	❑	❑	❑	❑	❑
Snow damages	❑	❑	❑	❑	❑
Drought damages	❑	❑	❑	❑	❑
Flooding damages	❑	❑	❑	❑	❑
Forest fire damages	❑	❑	❑	❑	❑
Increased competition from ground vegetation	❑	❑	❑	❑	❑
Reduced revenues from forestry	❑	❑	❑	❑	❑
Increasing interest rates	❑	❑	❑	❑	❑
Increasing property taxes	❑	❑	❑	❑	❑

40. Would you be willing to change your land-use to counteract climate changes? If that is the case, how?

(mark with one cross on each row)

	Definitely yes	Probably yes	Probably not	Definitely not	Do not know
Receive subsidies from the government for converting unforested land to forest land in order to store more carbon	❑	❑	❑	❑	❑
Receive payments from companies for converting unforested land to forest land in order to compensate for their emissions of carbon dioxide	❑	❑	❑	❑	❑
Receive subsidies from the government for fertilizing forest land in order to store more carbon	❑	❑	❑	❑	❑
Receive payments from companies for fertilizing forest land in order to compensate for their emissions of carbon dioxide	❑	❑	❑	❑	❑
Getting paid to provide land for establishment of wind power plants	❑	❑	❑	❑	❑
Establish wind power plants myself on my own land	❑	❑	❑	❑	❑
Modify forest management to store more carbon even if it would affect biodiversity negatively	❑	❑	❑	❑	❑
Grow forest on grazing land	❑	❑	❑	❑	❑
Grow forest on crop land	❑	❑	❑	❑	❑
Use grazing land for some other purpose	❑	❑	❑	❑	❑
Use crop land for some other purpose	❑	❑	❑	❑	❑

41. How would you react if up to three wind power plants were established on neighboring management units?

Very positive — Neutral — Very negative

42. How would you react if more than three wind power plants were established on neighboring management units?

Very positive — Neutral — Very negative

|----|----|----|----|----|----|----|----|----|----|

A few questions about extension services

43. Do you consult a forestry advisor (example of forestry advisors)?

❑ Yes ❑ No

44. If you answered yes to question 43, which organization do you usually engage?

(mark with one cross)

❑ Forest Agency (or equivalent)
❑ Forest owners association
❑ Example of an important company in the region

❑ Other organization: ________________________

45. If you answered no to question 43, why not?

(mark with one cross)

❑ I do not know whom to contact
❑ I do not need to contact an advisor

❑ Other reasons: _________________________

46. Do you discuss risks (such as those mentioned in question 39) in forestry with a forestry advisor?

❑ Yes ❑ No

47. Have you gotten advice or have you given advice about any of the following risks?

(mark with one cross for each event)

Events	Gotten advice from an advisor	Gave advice to another forest owner
Root rot damages	❑	❑
Bark beetle damages	❑	❑
Pine weevil damages	❑	❑
Browsing damages	❑	❑
Storm damages	❑	❑
Frost damages	❑	❑
Increased logging costs because of absence of ground frost	❑	❑
Snow damages	❑	❑
Drought damages	❑	❑
Flooding damages	❑	❑
Forest fire damages	❑	❑
Increased competition from ground vegetation	❑	❑
Reduced revenues from forestry	❑	❑
Increasing interest rates	❑	❑
Increasing property taxes	❑	❑
Other: ___________________________	❑	❑

48. Do you think that your forestry advisor knows enough to give you advice about the risks in the previous question?

❑ Yes
❑ Yes, for some of the mentioned risks, but not for: __________________________
❑ No

49. Was your forestry advisor your main source of information/advice regarding the risk of damages by storm/fire during a 5-year period before the storm/fire (date – date)?

❑ Yes ❑ No ❑ I do not have any forestry advisor

50. If you have answered yes to question 49, did you yourself bring up the question?

❑ Yes ❑ No

51. If you have answered no to question 49, who/what was your main source of information during that period?

❑ Relatives, neighbors or friends
❑ The former owner of my management unit
❑ The owner of a neighboring management unit
❑ The owner of a management unit that has many similarities with my management unit
❑ Books, journals, TV, radio, internet etc.

52. If your main source of information in question 51 was another person, did you yourself bring up the question?

❑ Yes ❑ No

53. If your main source of information in question 51 was another person, how do you think this person has gained his/her knowledge about forestry?

❑ Little or no knowledge about forestry
❑ Experiences from older generations or from own work
❑ Books, journals and magazines about forestry (specialist literature)
❑ Courses and meetings
❑ Forestry education of 1 year or more
❑ Do not know

A few questions about the situation <u>after</u> the storm/fire (date)

54. Have you, <u>after</u> the storm/fire (date) gotten concrete advice by your <u>forestry advisor</u> regarding the risk of damages by storm/fire?

❑ Yes ❑ No ❑ I have no advisor

55. If you have answered <u>yes</u> to question 54, did you yourself bring up the question?

❑ Yes ❑ No

56. If you have answered <u>no</u> to question 54, who/what was your main source of information regarding the risk of damages by storm/fire since the storm/fire (date)?

❑ Relatives, neighbors or friends
❑ The former owner of my management unit
❑ The owner of a neighboring management unit
❑ The owner of a management unit that has many similarities with my management unit
❑ Books, journals, TV, radio, internet etc.

57. If your main source of information in question 56 was another person, did you yourself bring up the question?

❑ Yes ❑ No

58. If your main source of information in question 56 was another person, how do you think this person has gained his/her knowledge about forestry?

❑ Little or no knowledge about forestry
❑ Experiences from older generations or from own work
❑ Books, journals and magazines about forestry (specialist literature)
❑ Courses and meetings
❑ Forestry education of 1 year or more
❑ Do not know

59. **Have you given advice to any other forest owner regarding the risk for storm/fire damages after the storm/fire (date)?**

❑ Yes ❑ No

60. **How has the storm/fire damages in (date) affected you emotionally?**

Very negative Not at all Very positive

61. **Are you worried about your private household economy because of the (storm/fire) damages after the storm/fire (date)?**

Very worried No change Not worried

62. **Do you think that the solidarity in the village/town where the management unit is located has changed after the storm/fire (date)?**

Decreased No change Increased

63. **Many European countries have given up clear-cut forestry in favor of continuous cover forestry without clear-cut areas. Would you be prepared to convert to continuous cover forestry?**

(mark with one cross)

❑ Yes, certainly ❑ No, scarcely
❑ Yes, perhaps ❑ No, definitely not ❑ Do not know

64. **What would you do if you had to make a very important decision regarding your forest management?**

(mark with one cross)

❑ I would ask for advice from the former owner of my management unit
❑ I would ask for advice from an owner of a neighboring management unit
❑ I would ask for advice from an owner that has a management unit similar to mine
❑ I would ask my forestry advisor (examples of forest advisors)
❑ I would do like I always have done
❑ I would assess what the future may bring and base my decision on that

⇨ What would that assessment be based on?

____

Some questions about different values in the forest

65. This question is about how you as a forest owner value different aspects of the forest and forest ownership. Please indicate how much value you assign to each aspect.

<u>0=no value, 10=very high value</u>

a. In which ways and to what degree does your forest have value for you as a resource for <u>timber production</u>? (0=no value, 10=very high value)

______ The financial gain from selling the timber

______ Having access to your own timber

______ The money I save by not having to buy timber

______ The things I construct from the timber

______ Contributing to the society by providing timber

______ Contributing to the country’s (national) finances

______ Other values: ___________________________________

b. In which ways and to what degree does your forest have value for you as a resource for <u>production of pulpwood</u>? (0=no value, 10=very high value)

______ The financial gain from selling the pulpwood

______ Contributing to the country’s (national) finances

______ Contributing to the access to paper for books/newspapers/magazines/etc. in the society

______ Other values: ___________________________________

c. In which ways and to what degree does your forest have value for you as a resource for bio energy production (including firewood)? (0=no value, 10=very high value)

______ The financial gain from selling the raw material

______ The financial gain from producing and selling my own bioenergy

______ The money I save from not having to by form other suppliers

______ The money I save by not having to buy raw material for bioenergy

______ The money I save by not having to buy raw material for bioenergy

______ The contribution of bio energy to the country’s national finances

______ The contribution of bio energy to decreased use of fossil fuels

______ Other values: ___________________________________

d. In which ways and to what degree does your forest have value for you as a __place for taking walks__? (0=no value, 10=very high value)

______ Improved health

______ Improved physical fitness

______ Relaxation

______ The sounds of the forest

______ Absence of disturbing sounds

______ Absence of disturbing impressions

______ Absence of other people

______ I appreciate the walking as such

______ Being present in the forest

______ The feeling of being in my own forest

______ Helps me think

______ Meeting the animals of the forest

______ Getting inspiration for artistic creation

______ Finding motives for artistic creation

______ Experiencing the beauty of the forest

______ Fresh air

______ Contributing to the public access to areas for recreation

______ Contributing to public health

______ Giving the public opportunities to get in contact with nature

______ Other values: ____________________________________

e. In which ways and to what degree does your forest have value for you as a __place for hunting__? (0=no value, 10=very high value)

______ Relaxation

______ Excitement

______ The good fellowship within the hunting group

______ Contributing to the country's (national) finances

______ Meat for my own consumption

______ My own financial gain from selling meat

______ Being able to eat meat from my own forest

______ My own financial gain from leasing hunting rights

______ I appreciate the hunting as such

______ Being present in the forest

______ Meeting the animals in the forest

______ Experiencing the beauty of the forest

______ Fresh air

______ Helps me think

______ Other values: ____________________________________

f. In which ways and to what degree does your forest have value for you as a <u>place for picking berries and mushrooms</u>? (0=no value, 10=very high value)

______ Relaxation

______ Health promotion

______ A way of spending time with the rest of the family

______ Berries/mushroom for my own consumption

______ The money I save by not having to buy berries/mushroom

______ The financial gain I get from selling the berries/mushroom

______ Contributing to the country's economy

______ The satisfaction from eating berries/mushroom from my own forest

______ Feels safer to eat berries/mushroom from my own forest

______ Appreciates the picking as such

______ Being present in the forest

______ Meeting the animals in the forest

______ Experiencing the beauty of the forest

______ Fresh air

______ Helps me think

______ Other values: ____________________________________

g. In which ways and to what degree does your forest have value for you as a <u>place for tourism</u>? (0=no value, 10=very high value)

______ My own financial gain from eco tourism

______ Contributes to the country's (national) finances by providing a place for eco tourism

______ Makes it possible for local people to make a living by eco tourism

______ Providing recreation opportunities for the public

______ Contributing to public health

______ Providing the public with opportunities to get in contact with nature

______ Contributing to increase people's appreciation of the values of the forest

______ Feel the pride that people want to visit my forest

______ Other values: ____

h. In which ways and to what degree does the <u>owning, administration and management of your forest</u> have value for you? (0=no value, 10=very high value)

______ My own financial gain from the forest

______ Contributes to the country's (national) finances

______ The possibility to choose silvicultural treatments based on my own motives

______ The possibility to manage the forestry business based on my own motives

______ The satisfaction of working in the forest

______ The satisfaction of working with forest economy/administration

______ The satisfaction of seeing the result of my work

______ Working with forestry is good for my physical health

______ Working with forestry is good for my mental health

______ Appreciates variation in my work

______ Appreciates working outdoors

______ Appreciates to be my own boss

______ Maintain family traditions

______ Wants to get interest from previously made investments

______ Wish to do something that will last after my life time

______ The status it gives me in the society

______ Financial security for my children

______ Ability to provide a secure environment for bringing up my children

______ Influencing my own and family's local environment

______ Other values: ____

i. In which ways and to which degree does your forest have value for you as <u>a provider of one or more of the following services</u>: clean water, protection against soil erosion and protection against air pollution? (0=no value, 10=very high value)

______ The financial gain I get from subsidies for providing one or more of these services

______ What one or more of these services give back to my forest

______ The contribution of one or more of these services to my agriculture/other business: …

______ The contribution of one or more of these services to the surrounding society

______ The contribution of one or more of these services to the future wellbeing of future generations in general

______ The contribution of one or more of these services to the future wellbeing of my own children

______ The contribution of one or more of these services to the ecosystem as such independently of what they contribute to humanity

______ Other values: ____

j. In which ways and to what degree does your forest have value for you as a <u>habitat for animals and plants</u>? (0=no value, 10=very high value)

______ The biodiversity of the forest

______ The forest as a home for a particular species that I value, viz ___________________

______ The knowledge that the species lives on my property

______ The knowledge that the species exists at all

______ The possibility to see/hear an individual of that species

______ Concern for the individuals of the species

______ The possibility to hunt/collect individuals of the species

______ The possibility to show the species to others on my property

______ The contribution the species has to the stability of the forest ecosystem

______ The contribution of the species to the economy of the forest (pest control, etc.)

______ Other values: ____

k. In which ways and to what degree does your forest have value for you as a <u>carbon sink</u>? (0=no value, 10=very high value)

______ Its contribution to counteract climate change

______ The value it provides my children by mitigating climate change

______ The financial gain I might get from companies paying me to compensate for their emissions

______ The financial gain I might get from government subsidies for keeping forest as a carbon sink

______ Other values: ___________________________________

66. Do you think that the strong demand for biofuel (from forest products) will be persistent over the next 10 years?

(mark with <u>one</u> cross)

❑ Definitely yes ❑ Probably yes ❑ Probably not ❑ Definitely not ❑ Do not know

67. Would you be willing to increase your acreage of forest land (if you have the possibility) to meet the possibly increasing demand for biofuel by:

(mark with <u>one</u> cross on each row)

	Definitely yes	Probably yes	Probably not	Definitely not	Do not know
Growing forest on <u>grazing land</u>	❑	❑	❑	❑	❑
Growing forest on <u>crop land</u>	❑	❑	❑	❑	❑
Growing forest on <u>other land</u>	❑	❑	❑	❑	❑
Convert <u>forest land</u> to energy crop production	❑	❑	❑	❑	❑

68. Assume that you have during several years invested time and money to keep the forest on your property well managed for timber production. Assume furthermore that you can improve the financial return by converting to production of raw material for bio-fuel production.

Is it more likely that you would continue manage the forest for timber production in the forest stands that you have started to manage for timber production, or that you would convert to production of raw material for bio-fuel production?

(mark with <u>one</u> cross on the scale)

Most likely that I would continue manage the forest stands for timber production	Most likely I would convert also these forest stands to production of raw material for bio-fuel production

Some question about you and your household

69. When were you born?

19___

70. Gender?

❑ Woman ❑ Man

71. What educations do you have?

❑ Elementary school or equivalent
❑ High school or equivalent
❑ University or equivalent
❑ Professional education or equivalent

72. How have you gained your knowledge about forestry?

❑ Little or no knowledge about forestry
❑ Experiences from older generations or from own work
❑ Books, journals and magazines about forestry (specialist literature)
❑ Courses and meetings
❑ Forestry education of 1 year or more
❑ Do not know

73. What was the total disposable income after taxes in your household during 2009?

(mark with one cross)

❑ 0 – 199 999 SEK ❑ 200 000 – 399 999 SEK ❑ 400 000 or more

74. How large share of the household's income (during 2009) came from the management unit?

(mark with one cross)

❑ Less than 5 % ❑ 16-25 % ❑ 51-75%
❑ 6-15 % ❑ 26-50% ❑ 76-100%

75. Are you a member of any of the following organizations?

❑ Forest owners association or equivalent
❑ Farmers association or equivalent
❑ I am not a member of any forest organization

76. **If the questionnaire was addressed to someone else than you, and you have filled it in, please state the gender and age here or the person that the questionnaire was addressed to.**

❑ Woman ❑ Man

Birthyear: 19____

If you want to comment or add something you can do that here:

____

____

____

____

____

____

____

____

____

____

____

____

____

____

____

____

____

Thank you for your participation in the survey!
