# Supplementary material for: Forest Owners' Response to Climate Change: University Education Trumps Value Profile
Source: PLoS One. 2016 May 25;11(5):e0155137. doi: 10.1371/journal.pone.0155137 (PMC4880312; doi:10.1371/journal.pone.0155137)
Supplement: S1 Table — (DOCX) [file pone.0155137.s006.docx]

**S1 Table. Questions assessing respondents' preferences for 95 services and benefits from the forest and the range and median score (0-10) assigned by respondents who reported having not studied or studied at university, based on a question reporting respondents' highest level of education per country (see Table 1). (n=766)**

| *Question* | | *Have no/have university education* | |
| --- | --- | --- | --- |
|  |  | Sweden n=351 | Germany n=415 |
|  |  | Range Median | Range Median |
| a. In which ways and to what degree does your forest have value for you as a resource for timber production? | 1. The financial gain from selling the timber | 0-10/0-10 9/8 | 0-10/0-10 10/10 |
|  | 2. Having access to my own timber | 0-10/0-10 5/4 | 0-10/0-10 9/8 |
|  | 3. The money I save by not having to buy timber | 0-10/0-10 3/2 | 0-10/0-10 6/4 |
|  | 4. The things I construct from the timber | 0-10/0-10 2/1.5 | 0-10/0-10 5/2 |
|  | 5. Contributing to the society by providing timber | 0-10/0-10 4.5/4 | 0-10/0-10 5/5 |
|  | 6. Contributing to the country’s (national) economy | 0-10/0-10 3/4 | 0-10/0-10 5/4 |
| b. In which ways and to what degree does your forest have value for you as a resource for production of pulpwood? | 7. The economic gain from selling the pulpwood | 0-10/0-10 8/8 | 0-10/0-10 6/5 |
|  | 8. Contributing to the country’s (national) economy | 0-10/0-10 3/3 | 0-10/0-10 3/2 |
|  | 9. Contributing to the access to paper for books/newspapers/magazines/etc. in the society | 0-10/0-10 4/4 | 0-10/0-10 2/2 |
| c. In which ways and to what degree does your forest have value for you as a resource for bioenergy production (including firewood)? | 10. The economic gain from selling the raw material | 0-10/0-10 5.5/6.5 | 0-10/0-10 8/7 |
|  | 11.The economic gain from producing and selling my own bioenergy | 0-10/0-10 1.5/2 | 0-10/0-10 2/0 |
|  | 12. The money I save from not having to by form other suppliers | 0-10/0-10 5/2 | 0-10/0-10 8/7 |
|  | 13. The money I save by not having to buy raw material for bioenergy | 0-10/0-10 1.5/1 | 0-10/0-10 8/7 |
|  | 14. The contribution of bioenergy to the country’s national economy | 0-10/0-10 2.5/4 | 0-10/0-10 4/4 |
|  | 15. The contribution of bioenergy to decreased use of fossil fuels | 0-10/0-10 5/5 | 0-10/0-10 5/6 |
| d. In which ways and to what degree does your forest have value for you as a place for taking walks? | 16. Improved health | 0-10/0-10 8/8 | 0-10/0-10 8/8 |
|  | 17. Improved physical fitness | 0-10/0-10 8/8 | 0-10/0-10 8/8 |
|  | 18. Relaxation | 0-10/0-10 9/9 | 0-10/0-10 8/9 |
|  | 19. The sounds of the forest | 0-10/0-10 8/8 | 0-10/0-10 7/7 |
|  | 20. Absence of disturbing sounds | 0-10/0-10 7/8 | 0-10/0-10 5/8 |
|  | 21. Absence of disturbing impressions | 0-10/0-10 6/7 | 0-10/0-10 5/8 |
|  | 22. Absence of other people | 0-10/0-10 5/5 | 0-10/0-10 5/5 |
|  | 23. I appreciate the activity as such | 0-10/0-10 8/8 | 0-10/0-10 5/5 |
|  | 24. Being present in the forest | 0-10/0-10 8/8 | 0-10/0-10 7/8 |
|  | 25. Helps me think | 0-10/0-10 6/7 | 0-10/0-10 7/7 |
|  | 26. Meeting the animals of the forest | 0-10/0-10 8/8 | 0-10/0-10 6.5/7 |
|  | 27. Getting inspiration for artistic creation | 0-10/0-10 1/2 | 0-10/0-10 0.5/0 |
|  | 28. Experiencing the beauty of the forest | 0-10/0-10 8/8 | 0-10/0-10 8/8 |
|  | 29. Fresh air | 0-10/0-10 9/9.5 | 0-10/0-10 9/10 |
|  | 30. Contributing to the public access to areas for recreation | 0-10/0-10 5/4.5 | 0-10/0-10 4/5 |
|  | 31. Contributing to public health | 0-10/0-10 5/4.5 | 0-10/0-10 5/5 |
|  | 32. Giving the public opportunities to get in contact with nature | 0-10/0-10 5/5 | 0-10/0-10 5/5 |
| e. In which ways and to what degree does your forest have value for you as a place for hunting? | 33. Contributing to the country’s (national) economy | 0-10/0-10 0/0 | 0-10/0-10 0/0 |
|  | 34. My own economic gain from selling meat | 0-8/0-10 0/0 | 0-10/0-10 0/0 |
|  | 35. My own financial gain from leasing hunting rights | 0-10/0-10 0/0 | 0-10/0-10 2/0 |
|  | 36. I appreciate the hunting as such | 0-10/0-10 2.5/3.5 | 0-10/0-10 5/4 |
| f. In which ways and to what degree does your forest have value for you as a place for picking berries and mushrooms? | 37. Relaxation | 0-10/0-10 8/7 | 0-10/0-10 5/5 |
|  | 38. Health promotion | 0-10/0-10 6/7 | 0-10/0-10 5/5 |
|  | 39. A way of spending time with the rest of the family | 0-10/0-10 6/7 | 0-10/0-10 3/3 |
|  | 40. Berries/mushroom for my own consumption | 0-10/0-10 7/7 | 0-10/0-10 4/4 |
|  | 41. The money I save by not having to buy berries/mushroom | 0-10/0-10 1.5/2 | 0-10/0-10 0/0 |
|  | 42. The financial gain I get from selling the berries/mushroom | 0-10/0-5 0/0 | 0-6/0-10 0/0 |
|  | 43. Contributing to the country’s economy | 0-10/0-5 0/0 | 0-10/0-10 0/0 |
|  | 44. The satisfaction from eating berries/mushroom from my own forest | 0-10/0-10 6/6 | 0-10/0-10 2/3 |
|  | 45. Appreciates the picking as such | 0-10/0-10 7/7 | 0-10/0-10 2/3 |
|  | 46. Being present in the forest | 0-10/0-10 8/8 | 0-10/0-10 5/5 |
|  | 47. Meeting the animals in the forest | 0-10/0-10 8/8 | 0-10/0-10 5/5 |
|  | 48. Experiencing the beauty of the forest | 0-10/0-10 8/8 | 0-10/0-10 6/7 |
|  | 49. Fresh air | 0-10/0-10 8/8 | 0-10/0-10 8/8 |
|  | 50. Helps me think | 0-10/0-10 5/5 | 0-10/0-10 4/5 |
| g. In which ways and to what degree does your forest have value for you as a place for tourism? | 51. My own economic gain from ecotourism | 0-10/0-10 0/0 | 0-10/0-10 0/0 |
|  | 52. Contributes to the country’s (national) economy by providing a place for ecotourism | 0-10/0-10 0/0 | 0-10/0-10 2/2 |
|  | 53. Makes it possible for local people to make a living by ecotourism | 0-10/0-8 0/0 | 0-10/0-10 4/3 |
|  | 54. Providing recreation opportunities for the public | 0-10/0-10 0/1 | 0-10/0-10 3/4 |
|  | 55. Contributing to public health | 0-10/0-10 0.5/1 | 0-10/0-10 5/5 |
|  | 56. Providing the public with opportunities to get in contact with nature | 0-10/0-10 3/2 | 0-10/0-10 5/5 |
|  | 57. Contributing to increase people’s appreciation of the values of the forest | 0-10/0-10 2/2 | 0-10/0-10 6/6 |
|  | 58. Feel the pride that people want to visit my forest | 0-10/0-10 2/1 | 0-10/0-10 2/2 |
| h. In which ways and to what degree does the owning, administration and management of your forest have value for you? | 59. My own economic gain from the forest | 0-10/0-10 7/6 | 0-10/0-10 10/10 |
|  | 60. Contributes to the country’s (national) economy | 0-10/0-10 3/4 | 0-10/0-10 5/5 |
|  | 61. The satisfaction of working in the forest | 0-10/0-10 8/8 | 0-10/0-10 9/8 |
|  | 62. The satisfaction of seeing the result of my work | 0-10/0-10 8/8 | 0-10/0-10 9/9 |
|  | 63. Working with forestry is good for my physical health | 0-10/0-10 8/8 | 0-10/0-10 8/7 |
|  | 64. Working with forestry is good for my mental health | 0-10/0-10 8/8 | 0-10/0-10 8/7 |
|  | 65. Appreciates variation in my work | 0-10/0-10 8/8 | 0-10/0-10 6/7 |
|  | 66. Appreciates working outdoors | 0-10/0-10 8/8 | 0-10/0-10 8/7 |
|  | 67. Appreciates to be my own boss | 0-10/0-10 8/8 | 0-10/0-10 8/8 |
|  | 68. Maintain family traditions | 0-10/0-10 6/5 | 0-10/0-10 8/8 |
|  | 69. Wants to get return from previously made investments | 0-10/0-10 6/5 | 0-10/0-10 8/7 |
|  | 70. Wish to do something that will last after my life time | 0-10/0-10 7/7 | 0-10/0-10 8/8 |
|  | 71. The status it gives me in the society | 0-10/0-10 1/0 | 0-10/0-10 4/3 |
|  | 72. Economic security for my children | 0-10/0-10 5/5 | 0-10/0-10 6/5 |
|  | 73. Ability to provide a secure environment for bringing up my children | 0-10/0-10 6/5 | 0-10/0-10 6/6 |
|  | 74. Influencing my own and my family’s local environment | 0-10/0-10 6/5.5 | 0-10/0-10 5/5 |
| i. In which ways and to which degree does your forest have value for you as a provider of one or more of the following services: clean water, protection against soil erosion and protection against air pollution? | 75. The economic gain I get from subsidies for providing one or more of these services | 0-10/0-10 1/2 | 0-10/0-10 2/2 |
|  | 76. What one or more of these services give back to my forest | 0-10/0-10 3/5 | 0-10/0-10 7/6 |
|  | 77. The contribution of one or more of these services to my agriculture/other business | 0-10/0-10 2/2 | 0-10/0-10 6/5 |
|  | 78. The contribution of one or more of these services to the surrounding society | 0-10/0-10 2/4 | 0-10/0-10 5/5 |
|  | 79. The contribution of one or more of these services to the future wellbeing of future generations in general | 0-10/0-10 3/5 | 0-10/0-10 8/8 |
|  | 80. The contribution of one or more of these services to the future wellbeing of my own children | 0-10/0-10 3/5 | 0-10/0-10 8/8 |
|  | 81. The contribution of one or more of these services to the ecosystem | 0-10/0-10 3/5 | 0-10/0-10 8/8 |
| j. In which ways and to what degree does your forest have value for you as a habitat for animals and plants? | 82. The biodiversity of the forests | 0-10/0-10 5/7 | 0-10/0-10 8/10 |
|  | 83. The forest as a home for a particular species that I value | 0-10/0-10 2/5 | 0-10/0-10 5/5 |
|  | 84. The knowledge that the species lives on my property | 0-10/0-10 5/5.5 | 0-10/0-10 5/5 |
|  | 85. The knowledge that the species exists at all | 0-10/0-10 5/5 | 0-10/0-10 5/5 |
|  | 86. The possibility to see/hear an individual of that species | 0-10/0-10 5/5.5 | 0-10/0-10 5/5 |
|  | 87. Concern for the individuals of the species | 0-10/0-10 5/5 | 0-10/0-10 3/2 |
|  | 88. The possibility to hunt/collect individuals of the species | 0-10/0-10 0/1 | 0-10/0-10 0/0 |
|  | 89. The possibility to show the species to others on my property | 0-10/0-10 0/1 | 0-10/0-10 1/0 |
|  | 90. The contribution the species has to the stability of the forest ecosystem | 0-10/0-10 2/4 | 0-10/0-10 5/5 |
|  | 91. The contribution of the species to the economy of the forest (pest control, etc.) | 0-10/0-10 0/4 | 0-10/0-10 4/3 |
| k. In which ways and to what degree does your forest have value for you as a carbon sink? | 92. Its contribution to mitigate climate change | 0-10/0-10 7/8 | 0-10/0-10 9/8 |
|  | 93. The value it provides my children by mitigating climate change | 0-10/0-10 7/5 | 0-10/0-10 9/10 |
|  | 94. The economic gain I might get from companies paying me to compensate for their emissions | 0-10/0-10 0/0 | 0-10/0-10 5/3 |
|  | 95. The financial gain I might get from government subsidies for keeping the forest as a carbon sink | 0-10/0-10 0/0 | 0-10/0-10 5/5 |
