# Supplementary material for: Forest Owners' Response to Climate Change: University Education Trumps Value Profile
Source: PLoS One. 2016 May 25;11(5):e0155137. doi: 10.1371/journal.pone.0155137 (PMC4880312; doi:10.1371/journal.pone.0155137)
Supplement: S2 Table — (DOCX) [file pone.0155137.s007.docx]

**S2 Table. Number of questionnaires distributed and returned with responses to the questions on preferences (S1 Table) per country.**

| ***Country*** | ***Distributed (n)*** | ***Returned and with preferences (n)*** | ***Return ratio with preferences (%)*** |
| --- | --- | --- | --- |
| **Sweden** | 683 | 351 | 51.4 |
| **Germany** | 652 | 415 | 63.7 |
| ***Total*** | *1335* | *766* | *57.6* |
