# Supplementary material for: Forest Owners' Response to Climate Change: University Education Trumps Value Profile
Source: PLoS One. 2016 May 25;11(5):e0155137. doi: 10.1371/journal.pone.0155137 (PMC4880312; doi:10.1371/journal.pone.0155137)
Supplement: S3 Table — (DOCX) [file pone.0155137.s008.docx]

**S3 Table. Number of clusters among respondents in scaling, and variance accounted for by these, by country.**

|  | *n* | *Variance accounted for before scaling (%)* | *Variance accounted for after scaling (%)* |
| --- | --- | --- | --- |
| Sweden | **7** | **58** | **64** |
| Germany | **7** | **54** | **59** |
