# Supplementary material for: Forest Owners' Response to Climate Change: University Education Trumps Value Profile
Source: PLoS One. 2016 May 25;11(5):e0155137. doi: 10.1371/journal.pone.0155137 (PMC4880312; doi:10.1371/journal.pone.0155137)
Supplement: S4 Table — (DOCX) [file pone.0155137.s009.docx]

**S4 Table. Missingness before imputation by question and country.**

| ***Country*** | ***Do you believe that the climate is changing to such an extent that it will substantially affect your forest? (%)*** | ***Have you experienced any extreme weather conditions or change in climate that you interpret as caused by long-term, global climate change? (%)*** | ***What is your highest education (%)*** |
| --- | --- | --- | --- |
| **Sweden** | 2.0 | 3.1 | 2.0 |
| **Germany** | 1.9 | 2.7 | 0.96 |
