# Supplementary material for: Forest Owners' Response to Climate Change: University Education Trumps Value Profile
Source: PLoS One. 2016 May 25;11(5):e0155137. doi: 10.1371/journal.pone.0155137 (PMC4880312; doi:10.1371/journal.pone.0155137)
Supplement: S5 Table — S.b. climate change—Strength of belief in the local effects of climate change, NU–No University education; U–University education; E–Environmentalists. The value profile Forest rejoicers was combined with Forest users and Sustainable forest users with Economic maximizers during model fitting because of quasi-complete separation (S4 Fig). The model was fitted to five imputed datasets using multinomial logistic regression. The mean null deviance = 910.6, the degrees of freedom for the null model = 1050, residual deviance = 888.5, and the residual degrees of freedom = 1044. The model fits the data significantly better than the null model (p = 0.0012). (DOCX) [file pone.0155137.s010.docx]

**S5 Table. Diagnostic statistics of model for predicting climate change risk perception in terms of strength of belief in the local effects of climate change by forest owners in Sweden based on education level and value profile.**

| *S.b. local effects climate change Predictor (base Probably not + Definitely not)* | *Value* | *SE* | *Z* | *p-value* |
| --- | --- | --- | --- | --- |
| Intercept: Yes, definitely | *-1.2* | *0.2* | *-5.39* | *7.1e-08* |
| Intercept: Yes, probably | *-0.2* | *0.2* | *-1.19* | *2.3e-01* |
| Intercept: I don’t know | *-1.0* | *0.2* | *-4.57* | *5.8e-06* |
| What education do you have (1=U, 0=NU): Yes, definitely | *0.9* | *0.4* | *2.41* | *1.6e-02* |
| What education do you have (1=U, 0=NU): Yes, probably | *0.7* | *0.3* | *2.30* | *2.2e-02* |
| What education do you have (1=U, 0=NU): Do not know | *0.3* | *0.4* | *0.579* | *5.6e-01* |
| Value profile (1=E, 0 other): Yes, definitely | *0.3* | *0.1* | *2.94* | *3.3e-03* |
| Value profile (1=E, 0 other): Yes, probably | *0.19* | *0.09* | *2.17* | *3.0e-02* |
| Value profile (1=E, 0 other): Do not know | *-0.0* | *0.1* | *-0.204* | *8.4e-01* |

S.b. climate change - Strength of belief in the local effects of climate change, NU – No University education; U – University education; E – Environmentalists. The value profile Forest rejoicers was combined with Forest users and Sustainable forest users with Economic maximizers during model fitting because of quasi-complete separation (S4 Fig). The model was fitted to five imputed datasets using multinomial logistic regression. The mean null deviance=910.6, the degrees of freedom for the null model=1050, residual deviance=888.5, and the residual degrees of freedom= 1044. The model fits the data significantly better than the null model (p= 0.0012).
