# Supplementary material for: Forest Owners' Response to Climate Change: University Education Trumps Value Profile
Source: PLoS One. 2016 May 25;11(5):e0155137. doi: 10.1371/journal.pone.0155137 (PMC4880312; doi:10.1371/journal.pone.0155137)
Supplement: S6 Table — S.b. climate change—Strength of belief in the local effects of climate change; NU–No University education; U–University education. The value profile Mushroom and berry pickers was combined with Diverse users and Conservationists with Economic maximizers during model fitting because of quasi-complete separation (S4 Fig). The model was fitted to five imputed datasets using multinomial logistic regression. The mean null deviance = 1093.8, the degrees of freedom for the null model = 1656, mean residual deviance = 1081.3, and the residual degrees of freedom = 1652. The model fits the data significantly better than the null model (p = 0.014). (DOCX) [file pone.0155137.s011.docx]

**S6 Table. Diagnostic statistics of model for predicting climate change risk perception in terms of strength of belief in the local effects of climate change by forest owners in Germany based on education level.**

| *S.b. local effects climate change*  *Predictor (base Definitely not)* | *Value* | *SE* | *Z* | *p-value* |
| --- | --- | --- | --- | --- |
| Intercept: Yes, definitely | *3.0* | *0.5* | *6.48* | *1.1e-10* |
| Intercept: Yes, probably | *3.3* | *0.5* | *7.24* | *6.5e-13* |
| Intercept: I don’t know | *2.0* | *0.5* | *4.29* | *1.8e-05* |
| Intercept: Probably not | *2.2* | *0.5* | *4.60* | *4.3e-06* |
| What education do you have (1=U, 0=NU): Yes, definitely | *-0.1* | *0.9* | *-0.134* | *0.89* |
| What education do you have (1=U, 0=NU): Yes, probably | *-0.9* | *0.9* | *-0.994* | *0.32* |
| What education do you have (1=U, 0=NU): Do not know | *-1.4* | *1.0* | *-1.38* | *0.17* |
| What education do you have (1=U, 0=NU): Probably not | *-0.1* | *0.9* | *-0.102* | *0.92* |

S.b. climate change - Strength of belief in the local effects of climate change; NU – No University education; U – University education. The value profile Mushroom and berry pickers was combined with Diverse users and Conservationists with Economic maximizers during model fitting because of quasi-complete separation (S4 Fig). The model was fitted to five imputed datasets using multinomial logistic regression. The mean null deviance=1093.8, the degrees of freedom for the null model=1656, mean residual deviance=1081.3, and the residual degrees of freedom= 1652. The model fits the data significantly better than the null model (p=0.014).
