# Supplementary material for: Forest Owners' Response to Climate Change: University Education Trumps Value Profile
Source: PLoS One. 2016 May 25;11(5):e0155137. doi: 10.1371/journal.pone.0155137 (PMC4880312; doi:10.1371/journal.pone.0155137)
Supplement: S7 Table — S.b. exp. climate change—Strength of belief in having experienced climate change; FU–Forest user value profile. The value profile Forest rejoicers was combined with Sustainable forest users and Economic maximizers with Conservationists during model fitting because of quasi-complete separation (S4 Fig). The model was fitted to five imputed datasets using multinomial logistic regression. The mean null deviance = 965.6, the degrees of freedom for the null model = 1400, the mean residual deviance = 954.3, and the residual degrees of freedom = 1396. The model fits the data significantly better than the null model (p = 0.024). (DOCX) [file pone.0155137.s012.docx]

**S7 Table. Diagnostic statistics of model for predicting climate change risk perception in terms of strength of belief in having experienced the effects of climate change by forest owners in Sweden based on value profile.**

| *S.b. exp. climate change Predictor (base Definitely not)* | *Value* | *SE* | *Z* | *p-value* |
| --- | --- | --- | --- | --- |
| Intercept: Yes, definitely | *0.9* | *0.3* | *2.51* | *1.3e-02* |
| Intercept: Yes, probably | *1.4* | *0.3* | *4.37* | *1.6e-05* |
| Intercept: I don’t know | *1.5* | *0.3* | *4.83* | *1.9e-06* |
| Intercept: Probably not | *2.4* | *0.3* | *7.99* | *1.6e-14* |
| Value profile (1=FU, 0 other): Yes, definitely | *-0.9* | *0.9* | *-0.965* | *3.4e-01* |
| Value profile (1=FU, 0 other): Yes, probably | *-0.2* | *0.7* | *-0.244* | *8.1e-01* |
| Value profile (1=FU, 0 other): Do not know | *-0.0* | *0.7* | *-0.0570* | *9.6e-01* |
| Value profile (1=FU, 0 other): Probably not | *-1.4* | *0.7* | *-1.87* | *6.1e-02* |

S.b. exp. climate change - Strength of belief in having experienced climate change; FU – Forest user value profile. The value profile Forest rejoicers was combined with Sustainable forest users and Economic maximizers with Conservationists during model fitting because of quasi-complete separation (S4 Fig). The model was fitted to five imputed datasets using multinomial logistic regression. The mean null deviance=965.6, the degrees of freedom for the null model=1400, the mean residual deviance=954.3, and the residual degrees of freedom= 1396. The model fits the data significantly better than the null model (p= 0.024).
